# Buffalo Academy of Medicine

**Published:** 1899-12

**Authors:** Albert J. Colton


### BUFFALO ACADEMY OF MEDICINE.

*Obstetrical Section, Tuesday Evening, September 26, 1899.*

REPORTED BY ALBERT J. COLTON, M. D., Secretary.

THE chairman, Dr. H. D. INGRAHAM, called the section to order at the regular hour of meeting. The minutes of the last regular session were read and approved, and the report of cases was taken up at once.

Dr. M. D. MANN presented specimens and reports of two cases:

Case I.—A large uterine fibroid, in which the cavity of the uterus was below the tumor. On cutting open the canal, a well developed ovum of about six weeks gestation was discovered. The fibrous growth was very large and growing rapidly, and this Dr. Mann ascribed to the pregnancy.

Case II.—That of a woman who was perfectly well, until three or four days ago, when she was suddenly seized with excruciating pain in the side, chills and pronounced shock. On examination, a tumor was found, evidently connecting with the ovary, and an opinion given that the symptoms were due to a twisting of its pedicle. At operation, a solid growth was found, which was connected with the ovary, and having a pedicle that was twisted three times. Doctor Mann said that he had never seen but one other solid tumor where there was a torsion of the pedicle, and that was a uterine fibroid.

Dr. Mann also read a letter from Dr. Coromilas, of Athens, Greece, the writer wishing to bring before the American medical profession, the fact that he was the originator of mechanical dilatation of the cervix and vulva during labor. Dr. C. also kindly sent his picture.

Dr. Thomas F. Dwyer made a motion, which was seconded and carried, that the letter be received and filed.

PAPERS.

The relation of gastric disturbances to gynecologic disease, by A. L. Benedict.

The author claimed that most of the inflammation and conditions in and around the uterus, ovaries, and the like, were accompanied by stomach symptoms, such as nausea, vomiting and pain. He cited several cases to prove these assertions.

The second paper was entitled Anesthesia, by F. W. McGuire, see page 325.

DISCUSSION.

Dr. Dunham complimented Dr. McGuire on his very able paper. He suggested that the room be quiet, when an anesthetic was being given, as the presence of talking friends kept the patient nervous. He also suggested that surgeons pay anesthetists larger fees and thus obtain good men.

Dr. T. F. Dwyer said he had read in a journal, some time ago, that the inhalation of vinegar, after an anesthetic, would prevent nausea, etc., and asked if the essayist had had experience with the same.

Dr. Grosvenor remarked that in England very little ether is used.

Dr. Mann said some of Dr. Benedict's cases do not illustrate the title of his paper very well. There is in some cases a close relation to the stomach and adnexa; but in the majority it is due to displacement. He cited several instances. In regard to anesthesia, he said he always used ether because it was the safer, but also said he had had five cases of death due to suppression of urine, Bright's disease being present in these cases. He suggested that we follow England, and have one experienced man in hospitals, whose duty it would be to anesthetise all patients for operation.

Dr. MILLENER suggested that in children, who are as a usual thing very much frightened, the anesthetiser attract the attention of the patient by diverting the mind from the anesthetic.

Dr. HARTWIG agreed entirely with Dr. McGuire, but added that he usually told patients what sensations they might expect, so that when they occurred, they would know it was a part of the procedure. He disagreed with Dr. Millener and said further it is absolutely impossible to distract the thoughts of children when taking chloroform, as they are almost frightened to death. On the other had, he usually follows Dr. Miner's advice, "when anesthetising children, push it as fast as you can."

Dr. CONGDON said that Dr. Benedict did not cite cases as clear as he should, and as to leucocytosis remarked that Dr. Krauss, who has just returned from Europe, quotes the Germans as saying that leucocytosis as a test for pus is of no more value than the urinary test for typhoid, and that any drain on the system will give the same results.

Dr. BENEDICT, in closing, said that he was aware that displacements of the uterus caused stomach symptoms more often than other surrounding troubles.

Dr. McGUIRE, in closing the discussion on his paper, said in reference to Dr. Millener's remarks, that in all the cases where he had given anesthetics to children, he had never known one to go to sleep without great struggle and outcry. He had no experience with vinegar and is guided by the rigidity of the eye-ball as to giving air or chloroform.

---

*Section on Medicine, October 10, 1899.*

REPORTED BY J. C. CLEMESHA, Secretary.

THE Medical Section of the Academy of Medicine held the first regular meeting in the Academy Parlors, Market Arcade, October 10, 1899. Meeting called to order 8.30 p. m. Dr. ELI H. LONG in the chair.

The minutes of the previous meeting were read and approved.

Dr. IRVING P. LYON read an exhaustive and interesting paper on Malaria, dwelling largely on the rôle of the mosquito in the evolution of the malarial parasite and upon the labors and writing of Manson, Ross and Italian investigators in that direction.

Dr. C. G. Stockton, in opening the discussion spoke on the question of change of type of fever from tertian or estivoautumnal to quartan or intermittent, and said the mosquito-malarial theory could account for such cases. He also brought up the question of the transmission of the malarial germ by the mosquito to its progeny and of the possibility of there being some other host than man, mentioning the rifeness of malaria in sparsely habited regions.

Dr. H. R. Hopkins wished to know the origin in nature and the locality, primarily, of the parasite of which the mosquito is the carrier.

Dr. Jack, of Depew, questioned the theory and referred to the action of certain ground gases upon the blood, causing alteration and disintegration of its elements.

Dr. Jewett stated that each year, for several years, he had seen cases of malaria in Buffalo, which with a few exceptions had been in the neighborhood of the Hamburg Canal.

Dr. A. A. Jones wished to know if the species anopheles existed in Buffalo and spoke of the transmission of other diseases by the common house-fly.

Dr. H. U. Williams paid a tribute to the various investigators and spoke of the difficulty, the patience and pains necessary for the technique of these experiments.

Dr. Lyon replied.

The meeting adjourned at 10.45 p. m. Members present, 51.

---

*Section on Pathology, Tuesday, October 17, 1899.*

Reported by FREDERIC H. MILLENER, M. D., Secretary.

THE second regular meeting of the section on pathology for the season was held on Tuesday, October 17, 1899.

The meeting was called to order by the chairman, Dr. A. G. Bennett, at 8.50 p. m. Thirty-three Fellows were present.

Minutes of the preceding meeting were read and approved.

The subjects for the evening were A case of poliencephalomyelitis in an adult, presenting the clinical picture of Landry's paralysis and terminating fatally in thirty-eight hours after the appearance of the first definite symptoms of motor disturbances in the legs, by Dr. Dewitt C. Sherman; and Chronic rhinitis and its relation to ocular affections, by Dr. Edmund A. Blaauw.

Dr. Sherman's patient was a medical student, aged 21, with clear personal and family history. One week before taken ill he received a dissecting wound, which was thoroughly cauterised and healed promptly. On Monday evening he called upon Dr. Sherman complaining of malaise and headache, thought to be due to constipation. Laxatives were administered. Next day nausea was present and lasted until Wednesday night. Wednesday afternoon the first definite symptoms of motor disturbances appeared in the legs. On Thursday, at 4 a. m., difficult respiration commenced, five hours later patient was compelled to assist respiration by pulling on iron bedstead with his arms. This gives us a picture of paralysis commencing in the feet, except the toes, and advancing rapidly up the body, so as to interfere with respiratory movements in about fourteen hours. He retained use of his arms until Thursday afternoon, when they became paralyzed. At 5 p. m. deglutition became difficult and at six he was unable to swallow. During that afternoon sensation was not even delayed. Electrical reactions were intact. All reflexes from shoulders down were lost. The intellect was clear. As artificial respiration was fast using up his strength, tracheotomy was performed in the evening at eight, in order to use an artificial respiration apparatus. Next morning at 1.40 he died. Gross post mortem signs were negative. The only microörganism found from collection of blood in all organs of the body was one similar to the bacillus coli communis. The pathological findings of the spinal cord and brain show an inflammatory process of the anterior cornu of cord and parts of brain with a well marked round-celled infiltration.

Dr. Sherman stated that the post mortem and bacteriological study was made by Dr. Herbert U. Williams. The historical pathology of the case, with remarks, was very comprehensively reported by Dr. William G. Spiller, of Philadelphia. The paper was then discussed by Drs. Putnam, Hartwig, Rochester and Long.

Dr. PUTNAM stated that it was the best presented case of its kind that he had ever had an opportunity of listening to, also, as Dr. Sherman stated, that the majority of the staff of the General Hospital were inclined to believe it hysteria, Dr. Cary being an exception, he declaring it to be Landry's paralysis. Dr. Putnam stated that he did not make a diagnosis. He also stated that a great many cases diagnosticated as Landry's paralysis were simply cases of multiple neuritis.

In connection with the case of Dr. Sherman, he reported a case at the General Hospital, which he had seen in consultation. This case differed from Dr. Sherman's in that there was gradual ascending paralysis, with loss of sensation and loss of control of bladder and bowels. The patient had almost total loss of faradic contractility. This was a case of affected ascending myelitis, and differs from Dr. Sherman's case in the fact that the sensory tracts of the cord were involved. The case ended fatally on the seventh day. He believed that this case was also the result of infection of the cord.

In closing, Dr. Sherman answered several questions which had been asked.

The next paper was that of Dr. BLAAUW. After a dissertation on the condition present in chronic rhinitis, the speaker mentioned the three ways in which the visible parts of the eye might be influenced.

(1) Through the secretion; (2) through the extention of the pathological process proper, the mucous membrane; (3) the indirect way.

1. The speaker pointed out a connection with eczema of the eyelids and what was called scrofulus ophthalmia, also the possibility of erysipelatous infection.

2. Hypertrophy extending in the nasal lachrymal duct, giving rise to stenosis with closure of the duct and the consequences. The speaker pointed out the importance of the enlargement of the arterior middle conchi. He also found same complaint in the hypertrophic stenosis of the duct as in unhealed chronic rhinitis. The results of treatment making the duct permeable is very satisfactory. The speaker developed his practice of not using large probes. He also mentioned Dr. Bettremieuz who found beneficial results in making the tear passages normal in tic douloureux. The principal point here is the headache. Too often the nose is overlooked in this relation. In closing Dr. Blaauw pointed out the necessity of the ophthalmologist being also a rhinologist.

The paper was discussed by Dr. Hinkel, Dr. Rochester, Dr. Cott and Dr. Bennett.

Dr. HINKEL stated that many of the varieties of hemicrania have origin in the nose, due to pressure symptoms, usually spur-like enlargements of the septum.

Dr. COTT stated that he did not believe disease of the duct was due to rhinitis as often as supposed.

Dr. BENNETT spoke of several cases in his practice in which phlyctenular conjunctivitis was caused by adenoid growth in the vault of the pharynx. In removing this condition the general health improved and so also the conjunctivitis.

